# Confronting the opioid epidemic: public opinion toward the expansion of treatment services in Virginia

**DOI:** 10.1186/s40352-019-0095-8

**Published:** 2019-07-23

**Authors:** Amy Kyle Cook, Nicola Worcman

**Affiliations:** 1 L. Douglas Wilder School of Government and Public Affairs, Virginia Commonwealth University, 1001 W. Franklin Street, Richmond, VA 23284 USA; 2 Substance Abuse Prevention, Treatment, and Policy, Virginia Commonwealth University, Richmond, VA USA

**Keywords:** Opioids, Public opinion, Community-based treatment, Recovery housing, Needle exchange

## Abstract

**Background:**

Public opinion polls have consistently shown Americans prefer treatment over arrest policies for opioid users. As the opioid epidemic remains a major health problem in the United States, it is important to determine the type of treatment policies the public would support. Theoretically, government should take into consideration the opinion of its constituents when deciding how to act. As such, the 2018 Virginia Commonwealth Public Policy Poll determined levels of support for the expansion of community-based treatment in one’s community.

**Results:**

Overall, the results showed 80% of Virginians (*n* = 788) supported the expansion of community-based treatment centers in their neighborhood, 69% supported the use of housing in their community, while less than half supported the provision of clean needles to IV drug users so they do not use dirty needles that could spread infection. Multivariate analyses revealed education, sex, and political party affiliation are significant factors in predicting support for the expansion of services.

**Conclusions:**

Given the lack of progress made by the government in reducing the supply and demand of drugs over the course of the war on drugs, it is time to move away from punitive policies to responsible and pragmatic approaches that include the expansion of community-based treatment.

## Background

Opioid use disorder remains a major health problem worldwide with 70% of the burden of disease attributable to the use of opioids (United Nations Office on Drugs and Crime [UNODC], 2017). The United States is responsible for 25% of drug related deaths worldwide, mostly from the use of opioids (UNODC, 2017). In the United States in 2017, there were over 72,000 fatal drug overdoses with over 47,000 the result of opioids (National Institute of Drug Abuse [NIDA], 2019). The effects of those numbers are profound considering more people die from the misuse of opioids than do from car accidents or violence (UNODC, 2017). Economists have estimated the United States’ economic burden of both the dependence of and fatal overdoses from heroin, prescription opioids, and synthetic opioids at $78.5 billion annually, including increased costs for health care, treatment, lost productivity, and criminal justice system involvement (Florence, Luo, Xu, & Zhou, 2016). Moreover, drug related deaths were attributed to a loss of .28 years in life expectancy (Dowell et al., 2017). Globally it was estimated that 17 million years of life lost were attributable to drug use in 2015 alone (UNODC, 2017).

The impact of drug abuse has far-reaching consequences in the lives of Americans. A recent public opinion poll showed that 30% of respondents felt drug abuse was a cause of trouble for their family (Gallup, 2018). When asked about the extent of the heroin problem in their area, 47% of respondents reported heroin was a very serious or somewhat serious problem with an additional 17% reporting it to be at crisis levels. Similar trends emerge with respect to prescription opioids. A number of public opinion polls show that addiction to prescription pain medication is a serious or major problem nationally (CBS News Poll, 2018; Gallup, 2018; Kaiser Family Foundation, 2016; Marist Poll, 2017; AP-NORC Poll, 2018; Pew Research Center, 2017) with 20 to 54% of Americans knowing someone who has suffered from opioid addiction (American Psychiatric Association, 2017; CBS News Poll, 2018; Marist Poll, 2017; Stat and Harvard T.H. Chan, School of Public Health, 2016). Moreover, roughly 20 to 25% of Americans reported knowing someone who died from prescription opioid use (Kaiser Family Foundation, 2017; Marist Poll, 2017; Stat and Harvard T.H. Chan, School of Public Health, 2016).

Public opinion polls have also compared preferences for treatment to criminal justice system responses. When faced with the choice to either increase access to treatment or impose stricter punishments and enforcement, Americans prefer policymakers increase access to treatment by 58% and 26%, respectfully (APA, 2017). Similarly, the preference of treatment over arrest for prescription opioids and heroin use was found in other polls (Cook & Brownstein, 2017; Pew Research Center, 2014). Despite the vast attention dedicated to the current opioid crisis, 43% of Americans believe the country is headed in the wrong direction as opposed to only 20% who feel the country is headed in the right direction in addressing the opioid crisis; 37% were not sure (APA, 2017). Furthermore, 37% of Americans feel the nation has lost ground in making progress with the drug problem (Gallup, 2017).

While the reasons are unknown as to why Americans reported feeling the country is headed in the wrong direction or that insufficient progress has been made in coping with the drug problem, it is important to consider the observe public support for the expansion of community-based treatment options. The following sections discuss various programs through which communities can utilize best practices to address the ever-growing substance abuse crisis as a public health problem. These include community-based treatment, recovery housing, and needle-exchange programs.

## Community-based treatment

Community-based treatment refers to comprehensive outpatient health care and psychiatric services offered in the community (United Nations Office on Drugs and Crime [UNODC], 2014). Based on a bio-psycho-social approach, community-based treatments are designed to help people with substance use problems develop the skills to manage their addiction in the community using a continuum of care model that reduces the need for residential and custodial services where possible (UNODC, 2014). According to UNODC (2014), community-based treatments are the most cost-effective method addressing drug use and dependence and have been associated with a reduction in hospital stays, emergency department visits, and criminal behavior.

Community-based treatments address a wide range of needs from detoxification through aftercare and involves the coordination of any number of health and social services needed to meet client’s needs to encourage change of behavior in the community (UNODC, 2014). Importantly, treatment services need to be available, accessible, affordable and evidence-based to deliver quality care for all people in need of support to help them reduce or stop the use of alcohol and other drugs (UNODC, 2014). Given that drug use is also associated with increased healthcare problems, particularly for people who inject drugs (PWID), expanding prevention and treatment opportunities and access is critical.

Currently, the United States offers a broad range of services based on evidence-based-programs designated for people who use drugs; however, availability and access to treatment for drug use remains a challenge. According to Substance Abuse and Mental Health Services Administration (2018), 20.7 million people in the United States were estimated to need substance use treatment, yet only 2.5 million received treatment. Some of the reasons for not receiving treatment include not being ready to stop using, a lack of healthcare coverage, not being able to afford the cost of treatment, believing seeking treatment would have a negative impact on employment, stigma from others, not knowing where to go for treatment, and not finding the type of treatment wanted (SAMSHA, 2018). Globally, only one out of six people with drug use disorders has access to treatment (UNODC, 2014, 2017).

## Recovery housing

Recovery housing or recovery residences are peer-run sober living environments that support individuals in their recovery from addiction or co-occurring mental health and substance use disorders (National Association Recovery Residences, 2012; Reif et al., 2014). Residents living in recovery homes receive a variety of services such as case management, therapeutic recreational activities, and peer support in order to improve functioning with the ultimate goal of integration back into the community (Reif et al., 2014). Safe and stable living environments are important to the recovery process especially for individuals with substance use disorders who need more structured care, typically after release from an inpatient facility (Reif et al., 2014). Often times those released from jail or prison are also in need of safe and stable living environments to continue recovery efforts. Blue and Rosenberg (2017) describe recovery housing as an essential component to the recovery process and without it, they contend recovery from addiction is unlikely, particularly given the challenges associated with low recovery capital. Low recovery capital refers to the challenges faced by those with substance abuse histories such as criminal history, low or no income, minimal work history, and poor credit resulting in difficulty in obtaining housing (Blue & Rosenberg, 2017).

Studies of recovery homes have shown a variety of improvements in residents functioning, employment, a reduction in substance use, lower rates of incarceration, improved family relationships, and a reduction in criminal activity (Jason, Aase, Mueller, & Ferrari, 2009; Jason, Davis, & Ferrari, 2007; Jason, Olson, Ferrari, & Lo Sasso, 2006; Mericle, Miles, & Way, 2015; Polcin, Korcha, Bond, & Galloway, 2010; Reif et al., 2014; Tuten, DeFulio, Jones, & Stitzer, 2012). Moreover, cost benefit analyses have shown that recovery housing saves nearly $29,000 per person considering the reduction in substance abuse, criminal activity, and incarceration (Lo Sasso, Byro, Jason, Ferrari, & Olson, 2012). Community-wide benefits such as reductions in homeless populations, a strengthened sense of community, and increased recovery capital in the community have also been noted in neighborhoods with recovery homes (Mericle & Miles, 2017; Polcin, Henderson, Trocki, Evans, & Wittman, 2012). Although studies of recovery houses are limited and not without criticism, research has shown that they are an important and preferred alternative to criminal justice involvement (Polcin et al., 2012).

## Harm reduction, including needle exchange programs

Harm Reduction is an umbrella term used to describe interventions and policies aimed to reduce the negative health consequences from substance abuse, particularly for those who inject drugs (Hawk et al., 2017; Logan & Marlatt, 2010) with the two primary goals of keeping people alive and protecting their health (Harm Reduction International, 2019). Harm reduction seeks to facilitate positive change regardless of how small or incremental and empower users to be primary agents of reducing the harms associated with their drug use (Harm Reduction Coalition, n.d). PWID are at greater risk for contracting HIV and Hepatitis C (Centers for Disease Control and Prevention, 2016). Considering that between 2000 and 2014 the number of acute infections of Hepatitis C among PWID doubled (Zibbell et al., 2018), needle exchange programs are an important component of harm reduction approaches as the sharing of needles increases the risk of transmission of blood-borne infections. The CDC (2016) estimates about one-third of PWID between the ages of 18–30 have Hepatitis C. Among older users, the rates are more concerning as 70–90% of older intravenous users have been diagnosed with Hepatitis C.

The use of needle exchange programs has demonstrated a reduction in both HIV and Hepatitis C infections (Abdul-Quader et al., 2013; Fernandes et al., 2017; Saab, Le, Saggi, Sundaram, & Tong, 2018). In addition to a reduction in the transmission of HIV and Hepatitis C, needle exchange programs are crucial to increasing access to other medical and social support services for PWID (European Monitoring Centre for Drugs and Drug Addiction, 2010; Hawk et al., 2017; Heimer, 1998; Wilson, Donald, Shattock, Wilson, & Fraser-Hurt, 2015; Wodak & Cooney, 2006). Although studies of needle exchange programs have shown promising outcomes for PWID, it is important to acknowledge that the widespread use of needle exchange programs is still limited (Abdul-Quader et al., 2013; Wilson et al., 2015). The limited use associated with needle exchange, despite their feasibility and cost-effectiveness, is likely a result of community resistance in which critics argue that harm reduction interventions may enable and encourage drug use and produce more risks and harm to the community (see Wodak & Cooney, 2006).

## Not in My Back Yard

The Not in My Back Yard (NIMBY) phenomenon is characterized by community resistance to having particular services such as housing developments, commercial establishments, health centers and other initiatives in one’s neighborhood (Furr-Holden et al., 2016; Kolla et al., 2017; Takahashi, 1997). Rather than being understood as a public good, community members oppose these facilities based on the assumption that characteristics of the clients that benefit from these services are objectionable (Davidson & Howe, 2014; Takahashi, 1997). According to Takahashi (1997), NIMBY is also related to the stigma associated with drug users, those with mental health problems, and the homeless. Lake (1993) described NIMBYism as an expression of needs and fears of community members.

Communities affected by NIMBYism may constitute an important barrier to not only the implementation but the continuing existence of health services such as drug treatment centers, housing, and needle exchange programs targeting PWUD (see Furr-Holden et al., 2016). Concerns related to property values, community safety, neighborhood identity, condoning and increasing drug use, and an increase in crime and violence have been cited as reasons residents have opposed services in their neighborhood (Davidson & Howe, 2014; Furr-Holden et al., 2016; Knopf, 2016; Kolla et al., 2017; Marx et al., 2000; Polcin et al., 2012). Marx et al. (2000) did not find a statistically significant difference in drug related offenses after the implementation of a needle exchange program. In a study examining whether there was an increase of violence near drug treatment centers as compared to the violence around convenience stores, corner stores, and liquor stores, Furr-Holden et al. (2016) found no statistical evidence that the presence of a drug treatment center attracted violent crime.

Similarly, in Sydney, Australia, researchers did not find that theft and robbery incidents increased around a medically supervised injection site (MSIC Evaluation Committee, 2003). Though the literature has not empirically shown a significant increase in crime, nonetheless, community members concerns related to NIMBYism are important considerations for planners (see Takahashi, 1997). As stated by Furr-Holden et al. (2016), “NIMBYism poses a significant threat to vital behavioral health services being located in communities” (p. 22).

Given the role NIMBYism plays regarding the inclusion of health services for people who use drugs (PWUD) coupled with previous public opinion polls that show Americans overwhelmingly support treatment over arrest policies, this study was designed to examine specific levels of public support for the expansion of community-based treatment services, recovery housing, and needle exchange programs in the respondent’s community.

## Methodology: a statewide public opinion poll

Given the importance of public opinion on the policy making process, the 2018 Commonwealth Public Policy Poll^1^ measured levels of support for the expansion of treatment services given the surge in opioid-related deaths in Virginia. In 2017, 1227 Virginians died of opioid overdoses that involved prescription pills, heroin, and fentanyl; more than half of those deaths were caused by fentanyl (Cammarata, 2018). Fentanyl is a synthetic opioid that is 100 times more potent than morphine and 50 times more potent than heroin (Drug Enforcement Administration (DEA), 2017). Between 2015 and 16, Virginia experienced a statistically significant increase in fentanyl related deaths (CDC, 2018). More specifically, the CDC (2018) reports that in 2015 there were 270 fentanyl related deaths in Virginia whereas in 2016, 648 deaths were attributed to illicitly manufactured fentanyl. Nationally, there was a 100% increase in fentanyl deaths from 2015 to 2016 (CDC, 2018). Given the significant increases in fentanyl related deaths, evidence suggests that the nature of the opioid crisis has evolved from prescription pills and heroin to illicitly manufactured fentanyl, causing the death toll to drastically increase.

For the Commonwealth Poll, between December 8–26, 2017, Issues and Answers Network conducted 788 telephone interviews with adult residents in the 5 regions in Virginia using random digit dialing. Soft quotas were implemented for gender and region. Two distinct sampling frames were used for wireless (*n* = 396; 50.3%) and landline phones (*n* = 392; 49.7%). Interviews were administered in English. The sampling margin of error is +/− 3.49 percentage points (95% confidence interval). Table 1 shows the demographic characteristics of the sample that comprises 52.3% females, 73% White, 2.8% Hispanic, with most respondents having post-secondary education (78%), and politically identifying as Democrat (33%), Republican (25%), and Independent (33%).

**Table 1:** Demographic characteristics of respondents (*N* = 788)

Characteristic	Sample Percentage
Sex	
Male	47.7
Female	52.3
Race	
White	73.4
Minority	21.1
Ethnicity	
Hispanic	2.8
Non-Hispanic	94.8
Education	
High school graduate or less	19.8
Post secondary education	77.8
Political Party Affiliation	
Democrat	33.0
Republican	24.5
Independent	33.1

Note. 5.6%, 2.4%, and 2.4% of the sample did not know or refused to identify race, ethnicity, and educational attainment, respectively. As for political party affiliation, 9.4% of the sample identified as something else, did not know, or refused to identify their party affiliation

### Measures

To determine whether or not Virginians support the expansion of treatment services, the following vignette was read to respondents: “In November 2016, the State Health Commissioner declared a public health emergency because of the opioid crisis. A public health approach recognizes the need to reduce the harms associated with drug use to both the individual user and the public through the expansion of treatment services.” Would you support or oppose

- The expansion of community-based treatment centers in your community?
- The use of housing in your community for those in recovery?
- Providing clean needles to IV drug users in your community so they don’t use dirty needles that could spread infection?

## Results

**Fig. 1:**
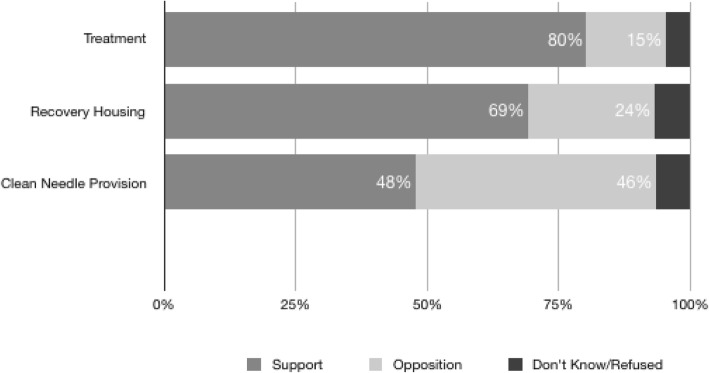
Support for Strategies to Combat the Opioid Crisis

Overall, as shown in Fig. 1, 80% of Virginians supported the expansion of community-based treatment centers in their neighborhood, 69% supported the use of housing in their community, with less than half (48%) supporting the provision of clean needles to IV drug users so they do not use dirty needles that could spread infection. While the expansion of treatment centers and recovery housing is high, support for providing clean needles to users is much lower. Further means testing shows significant differences between race and ethnicity for the expansion of community-based treatment centers as well as race for the support for recovery housing. Specifically, significant differences were found between Whites (M = 1.54, SD = 1.62) and minorities (M = 1.23, SD = .83) in support for the expansion of community-based treatment centers [t(742) = 3.305, *p* = .000] and between Hispanics (M = 1.05, SD = .213) and non-Hispanics (M = 1.49, SD = 1.503) for the expansion of community-based treatment centers [t(767) = − 6.212, *p* = .012]. With respect to support for recovery housing, there are significant differences between Whites (M = 1.75, SD = 1.81) and minorities (M = 1.55, SD = 1.42) [t(742) = 1.469, *p* = .04]. There were no significant differences found between sex, education level, or political party affiliation.

Three logistic regression models were estimated using those questions as dependent variables (dummy coded as 0 = opposition and 1 = support). Demographic variables were coded in such a way as to reflect differences in policy perspectives: sex (0 = male, 1 = female), race (0 = White, 1 = minority), ethnicity (0 = non-Hispanic, 1 = Hispanic), education level (0 = high school graduate or less, 1 = post secondary education), and political party affiliation (1 = Democrat, 2 = Republican, 3 = Independent). Table 2 presents the results of the logistic regression models that examine factors associated with support or opposition for the expansion of community-based treatment services (a), recovery housing (b), and needle-exchange programs (c).

**Table 2:** An examination of the factors regarding Virginians support for the expansion of community based treatment services, recovery housing, and needle exchange programs

Variables grams	Model 1 Community based treatment		Model 2 Recovery housing		Model 3 Needle exchange programs	
	b(SE)	OR	b(SE)	OR	b(SE)	OR
Education	.656(.240)	1.927**	.357(.201)	1.430	.112(.182)	1.119
Sex	.614(.240)	1.848*	.678(.196)	1.969**	.178(.172)	1.194
Democrat	.383(.334)	1.466	.568(.258)	1.765*	.586(.215)	1.798**
Republican	−.700(.278)	.496*	−.272(.234)	.762	−.761(.220)	.467**
Race	−.284(.323)	.752	−.319(.257)	.727	−.309(.218)	.734
Hispanic	.577(.772)	1.780	1.104(.670)	3.015	1.999(.582)	7.383**
Cox & Snell R^2^	.047		.051		.092	
Nagelkerke R^2^	.086		.077		.123	
Chi-square	31.035***		33.157***		58.766***	

*p* < .000***, *p* < .01**, *p* < .05*

Model 1 was statistically significant (chi square = 31.035, *p* = .000) and explained 9% of the variation in supporting the expansion of community-based treatment centers in one’s community. Three significant relationships emerged: education (*p* = .006), sex (*p* = .01), and identifying as a Republican (*p* = .01). Post secondary education and sex were positively associated with supporting the expansion of community-based treatment in one’s community while identifying as a Republican was negatively associated with expanding services. More specifically, having post-secondary education and being female increased the odds of supporting the expansion of community-based treatment services by 93 and 85%, respectively. Identifying as a Republican decreased the odds of supporting the expansion of community-based treatment services by 50%. Conversely, Republicans were more likely to oppose rather than support the expansion of community-based treatment services.

Model 2 explained 7% of the variation in support for the use of housing in their own community; the model was statistically significant (chi square = 33.157, *p* = .000). Identifying as a Democrat (*p* = .02) and female (*p* = .001) were revealed as statistically significant factors supporting the use of housing in their community. Being female increased the odds of supporting housing by 97% while identifying as a Democrat increased the odds by 76%. It is worth noting that the education variable approached significance (*p* = .07).

Model 3 examined support for providing clean needles to IV drug users to reduce the spread of infection. The model was statistically significant (chi square = 58.766, *p* = .000) and explained 12% of the variation in supporting the provision of clean needles to users. Three significant relationships developed: being Hispanic (*p* = .001), and identifying as both a Democrat (*p*. = .006) and Republican (*p* = .001). Being Hispanic increased the odds of supporting the provision of clean needles to IV drug users by 638% and identifying as a Democrat increased the odds of support by 80%; however, identifying as a Republican reduced the odds of support for the provision of clean needles to IV drug users by 53%. The following section will provide a discussion of these results as well as policy implications.

## Discussion

Previous work has shown the importance of public opinion polls on policy making (Cook & Brownstein, 2017). As the opioid problem evolves and even worsens, determining public support (or opposition) for the expansion of treatment services in one’s own community is a critical component for policy makers given the devastating impact of the opioid epidemic. Not only did these measures examine support for the expansion of treatment services, it did so in the context of asking about specific support “in your community”, an important inclusion considering the NIMBY phenomenon. The current study found that Virginians overwhelmingly supported the expansion of treatment centers and recovery housing in their own community although much lower levels of support were found for the provision of needle exchanges to IV drug users (further discussion provided later).

When examining the factors that indicated support for the expansion of treatment in one’s own community, higher levels of education, that is being educated beyond high school and being female were statistically significant factors whereas identifying as a Republican showed opposition to the expansion of treatment services. As for the expansion of recovery housing “in your community”, significant determinants were being female and identifying as a Democrat. The significance of being female likely reflects the 260% increase in drug overdose deaths among women aged 30–64 between 1999 and 2017 (VanHouten, Rudd, Ballesteros, & Mack, 2019). There are a variety of factors that explain the increase in overdose deaths illustrating the unique experiences faced by women who use drugs. As compared to men, women who use drugs become addicted sooner, show different impacts on the brain, and are more likely to relapse, overdose, attempt suicide, report adverse childhood experiences, and have mental and physical health problems (Bloom, Owen, & Covington, 2003; Darke, Campbell, & Popple, 2012; Felitti et al., 1998; NIDA, 2018a).

At the turn of the twentieth century, the first wave of the opioid epidemic, women were front and center to the marketing of and prescribing of opioids - they were prescribed opioids for menstrual cramps and hysteria (Terplan, 2017). While the reasons for prescribing opioids may have changed, the iatrogenic nature of the current opioid crisis parallels that of the first (Kolodny et al., 2015; Terplan, 2017). Understanding the experiences and challenges faced by women are paramount to adequately addressing and treating their substance abuse needs; substance abuse programming and treatment should reflect those differences. The results also reveal the importance of education among its citizenry. Higher levels of education may be the foundation for a more informed understanding of addiction and treatment needs.

Expanding community-based treatment services and recovery housing are essential components in the recovery process and fills a service gap (Blue & Rosenberg, 2017; Substance Abuse and Mental Health Services Administration, 2013). The current study underscores the importance of expanding the accessibility of both community-based treatment and recovery housing for those with substance abuse histories because without both, users will likely find recovery unattainable given the challenges with respect to low recovery capital (Blue & Rosenberg, 2017). As previously noted, community-based treatment programs are cost-effective as compared to hospital emergency room usage and incarceration (UNODC, 2014). Moreover, participants in recovery homes have shown improvements in social and family functioning, employment, and reductions in criminal behavior, substance abuse, and incarceration (Jason et al., 2006, 2007, 2009; Mericle et al., 2015; Polcin et al., 2010; Reif et al., 2014; Tuten et al., 2012).

With respect to providing clean needles to IV drug users, being Hispanic and identifying as a Democrat significantly predicted support; conversely, identifying as a Republican significantly indicated opposition. The robustness of political affiliation across models is interesting given recent bipartisan political support for dealing with the current crisis. Despite the current study’s findings among the general population, support by Republican politicians for sensible and pragmatic policies such as harm reduction approaches, including needle exchange programs is growing in response to the current crisis (Nadelmann & LaSalle, 2017).

## Virginians’ opinions: harm reduction and needle exchange programs

Given the public’s high levels of support for the expansion of community-based treatment and recovery housing, lower levels of support for the provision of needle exchanges may be explained by a number of factors such as lack of education about the scope of such programs considering their benefits to users, stigma associated with people who use and inject drugs, the NIMBY phenomenon. It may also be that citizens distinguish community-based treatment and recovery housing from needle exchange programs because the former helps users stop using drugs whereas needle exchange programs allow drug use to continue, although more safely. Regardless, the benefits of needle exchange programs cannot be overstated - they reduce the harms associated with opioid use by offering clean syringes and needles as well as other injection equipment and safe disposal containers, offer HIV and hepatitis testing, provide overdose prevention, educate users about safe injecting practices, and offer tools to prevent HIV and other sexually transmitted diseases that includes condoms and counseling. Most notably, exchange programming includes referrals to substance abuse treatment, medical and mental health care, and other social services (CDC, 2017). Research has shown that exchange programs are compatible with the goals of treatment and do not increase drug use or crime (CDC, 2017; Furr-Holden et al., 2016; Hagan et al., 2000; Heimer, 1998; Marx et al., 2000). Furthermore, needle exchange programs save on costs associated with healthcare while participants of needle exchange programs are five times more likely to enter into treatment than those that are not participants of exchange programs (CDC, 2017; Hagan et al., 2000).

With respect to model 3 and examining support for needle exchange programs, it is important to note the small percentage of Hispanics included in the study (less than 3%). However, the significance of identifying as Hispanic was initially an unexpected finding as research has shown Hispanics are less likely to have a SUD compared to individuals born in the United States (Salas-Wright, Vaughn, Clark Goings, Córdova, & Schwartz, 2018). It should be noted however, that Salas-Wright et al. (2018) suggest that the lower rates of Hispanics self-reports of substance use may be related to immigration status and fear of deportation. Nevertheless, the significance of identity as Hispanic could be related to two hypothesis.

First, health outcomes associated with substance use among Hispanics may explain the significant support for needle exchange programs. For example, intravenous drug use among Hispanics accounted for 19% of the cases diagnosed with HIV in 2015 (CDC, 2016). Moreover, recent changes in opioid related deaths among Latinos may explain support. Between 2013 and 2015 Hispanics made up 2 % of opioid related deaths in Virginia; that number rose to 3 % in 2016 (Kaiser Family Foundation, 2018). Other states such as New York and Massachusetts have also experienced increases in deaths among Hispanics. In Massachusetts the death rate among Hispanics doubled between 2014 and 16 (twice the rate of other groups) while in New York, over half of the deaths were attributed to fentanyl (Bebinger, 2018; Frisneda, 2017). Nationally, opioid related deaths among Latinos rose 35% while synthetic deaths increased by 183%, between 2015 and 2016 (as cited in Rosello, 2018). Support among Latinos for needle exchange programs may also reflect broader changes in attitudes or moral values which occurs as part of the acculturation process to the American culture especially for Latinos in the United States (Flórez et al., 2015). Florez et al. also explain that the escalating violence in Latin American countries may shape attitudes toward drug use. Second, the growing levels of substance use among Hispanics might be seen as a maladaptive coping strategy among emerging adults (Allem, Soto, Baezconde-Garbanati, & Unger, 2015).

The considerable focus on harm reduction, including needle exchange programs are important considerations because Virginia has been identified as a jurisdiction that is experiencing or is at-risk of experiencing significant increases in HIV or Hep C as a result of intravenous drug use with 8 localities in particular considered vulnerable (Van Handel et al., 2016). In response to these high rates of HIV and Hepatitis C in Virginia, in July 2017, House Bill 2317 authorized needle exchange programs to operate in 55 pre-identified localities; however, to date there are only two programs operating in the state. One in Wise County, where the rate of Hepatitis C is double that of the state rate (Friedenberger, 2018) and the other that opened in Richmond in November, 2018 (Balch, 2018). The law requires that entities applying to operate needle exchange programs in those pre-approved localities must have the support of both local law enforcement and the health department (Virginia House Bill 2317, 2017).

Garnering support from local law enforcement agencies may be more problematic than originally thought given that only two applications have been approved to operate such a program (Friedenberger, 2018). It is not difficult to understand the reticence of police to support needle exchange programs given the enforcement aspect of their job. After all, police serve to enforce laws, which means possession of paraphernalia laws are likely enforced when police come across a user with drug laced needles or other injection equipment. Although police and other abstinence groups may oppose such programs, they are an important partner to have in developing programs to avoid interference and client harassment with exchange programs (see Beletsky, Grau, White, Bowman, & Heimer, 2011; Strike, Myers, & Millson, 2004).

Though it has been noted that police and other groups have complicated the establishment of needle exchange programs, over time they have been swayed to support such programs based on the scientific evidence of their effectiveness (Strike et al., 2004). As Strike et al. (2004) noted, a police officer on the committee initially was not supportive of the program and wanted to make sure it never happened but after learning about the benefits to the users of the program, he eventually became an advocate of the program. More recently, in North Carolina, a border state just south of Virginia, a study of police officers indicated that officers were supportive of decriminalization of syringes to reduce Hepatitis C and HIV and believed that decriminalization would be good for the community as well as law enforcement (Davis et al., 2014). Given these concerns, collaborative efforts that include voices of opposition coupled with evidence from the scientific community highlighting the effectiveness of needle exchange programs while debunking concerns such as condoning drug use and increasing crime rates are critical to their success. The CDC (2016) recommends health departments should work with police and local leaders to expand needle exchange programs.

Despite the fact that addiction is defined as a chronic disease of the brain (NIDA, 2018b), many Americans believe addiction is the result of choice, a lack of willpower or discipline, character defect, bad parenting, or they outright blame users (AP-NORC Poll, 2018; Kaiser Family Foundation, 2016; Palamar, 2013). This lack of understanding of addiction clouds perceptions and contributes further to addiction related stigma. By simplifying addiction to a mere choice, we ignore both the medical and environmental factors associated with addiction via the disease model (see McLellan, Lewis, O’Brien, & Kleber, 2000). Interviews with participants in treatment have provided evidence that understanding addiction from a genetic framework would decrease stigma (Dingel, Ostergren, Heaney, Koenig, & McCormick, 2017).

In a study that examined vignettes regarding individuals with untreated versus treated mental health and opioid addiction, differences were noted (McGinty, Goldman, Pescosolido, & Barry, 2015). Respondents who received more information regarding successful treatment showed improved attitudes towards mental illness and addiction, suggesting that portrayals of successful treatment may mitigate negative attitudes held by society (McGinty et al., 2015). Moreover, personalizing accounts of those directly impacted by opioids may be one of the best ways to overcome addiction related stigma. In fact, the CDC (2019) explains that the use of evidence-based campaigns work to increase awareness by humanizing those that suffer from addiction and by extension, address and reduce stigma and increase access to services including harm reduction strategies. The implications of stigma can be far reaching and as Olsen and Sharfstein (2014) so poignantly stated, “this stigma is impeding progress in reducing the toll of overdose” (1393).

## Policy implications

The importance of political influence and persuasion cannot be overstated. Just as politicians had an influence on public opinion during the get tough on crime movement in the 1980s and 1990s, Wozniak (2016) asserts that the public can be reassured by politicians that the programs they endorse are effective. Considering their powers of persuasion it is likely that politicians have the clout to influence the general public and the law enforcement community on the benefits of community-based resources and harm reduction approaches. As research shows, community-based treatments and harm reduction strategies such as needle exchange programs are cost-effective and improve the lives of PWUD (Wilson et al., 2015).

Given the sentiment of law enforcement that “We can’t arrest our way out of this problem” (see Truong, 2017), law enforcement would benefit from accurate education from the public health and scientific communities about the advantages of and opportunities for non-arrest pathways to treatment (see the Police Assisted Addiction and Recovery Initiative [PAARI], n.d; Police, Treatment, And Community Collaborative, n.d; Seattle’s LEAD Program, 2018; Cloud & Davis, 2015). Non-arrest pathways are important tools for police in that they create opportunities to respond to the demand side of the supply and demand drug markets (see PAARI).

Since the latter part of the twentieth century, the war on drugs has primarily been the paradigm in which our government has responded to eradicating drugs and punishing offenders (Neil, 2014). The punitive focus on zero-tolerance policies, increased penalties, and incarceration became a substitute for treatment, leaving the needs of drug users unmet (Neil, 2014). Even in the realm of corrections, mainly prisons, the focus on offenders was more about punishment rather than rehabilitation (Balboni, 2013). Interestingly enough, a National Association of Chiefs of Police (2005) survey found that 82% of chiefs and sheriffs did not believe the war on drugs has been successful in reducing the use of illegal drugs. This suggests that police - those at the forefront of the drug crisis - have great insight to the limited nature and failures of the war on drugs. Perhaps it is the cost of the war on drugs, estimated at 1 trillion dollars since the early 1970s (Pearl, 2018), coupled with the current opioid crisis that has resulted in a shift away from the war on drugs to a public health approach with increasing momentum (Pope, Davis, Cloud, & Delaney-Brumsey, 2017).

This is not suggested that arrest policies may not be necessary at times but rather thinking about how public health initiatives over time improve lives of some of our most vulnerable citizens and how the criminal justice system can serve as a pathway to treatment (see Pope et al., 2017). As police and other criminal justice officials are increasingly having to deal with issues of behavioral health such as drug use, mental illness, and other social ills, creating opportunities that advance health and justice are necessary (Cloud & Davis, 2015). As Cloud and Davis (2015) assert, “The lack of adequate community-based mental health treatment, housing options, and harm reduction services across the United States underlies many of the challenges that police, courts, and jails encounter when interacting with people with complex health needs” (p.20).

To advance public safety, criminal justice, and public health, those working to ameliorate the effects of addiction must continue to educate the public and partner with public safety agencies. By reshaping our understanding of addiction and treatment, it not only benefits PWUD but it also serves to enhance public safety. Future studies regarding the perceptions of law enforcement’s support for needle exchange programs and those deserving of diversion efforts are needed.

## Limitations

Several limitations are noted. First, in terms of generalizability, the results from the current study may be generalizable to other states with similar levels of opioid problems and demographics as that of Virginia. Though this study showed that Democrats were more supportive of needle-exchange programs, it is important to note that Republican leaning states have passed legislation authorizing needle exchange programs to combat the transmission of Hepatitis and HIV (Kaiser Family Foundation, 2019). At the top levels of the federal government, the Secretary of Health and Human Services Alex Azar, a Republican, also supports the use of needle exchange programs (Azar, 2019).

Although the Commonwealth of Virginia is a “blue state” with Democratic leadership in the executive branch of government, political orientation is complicated as it relates to the expansion of services, particularly in Virginia where the support of law enforcement is necessary by law to establish needle exchange programs. In bordering North Carolina, a number of police chiefs and sheriffs support syringe programs because of the positive impacts on communities and intravenous drug users (North Carolina Harm Reduction Coalition). Regardless of political orientation, the findings underscore the need for government, health departments, local service providers, including law enforcement, to work together to implement evidence-based strategies that expand services.

Second, the time-dimension is cross-sectional in nature. Third, the study is limited in terms of explanatory factors and does not consider factors beyond demographics that may explain levels of support (or lack thereof) for treatment strategies. The inclusion of additional survey items such as knowing someone with a substance abuse problem or personal experience with substance abuse may help explain levels of support. Fourth, it should be noted that the questions were broadly worded to account for a lack of prior knowledge of such approaches. For example, the question pertaining to the provision of clean needles to PWID was initially worded as support or opposition for NEP; however, pilot testing revealed some confusion on that question as respondents were not familiar enough with the term “needle exchange” and therefore unable to answer the question. As such, the question was reworded to include an explanation of the concept of needle exchange programs: Would you support or oppose “*Providing clean needles to IV drug users so they don’t use dirty needles that could spread infection?*” Nevertheless, the data provides valuable insight for communities, health departments, law enforcement agencies, and politicians as it relates to the expansion of community based treatment.

## Conclusion

Given the iatrogenic nature of the opioid crisis, comprehensive education that includes scientific information is needed so that the public can understand the nature of addiction. The failure to understand addiction means we also fail to respond appropriately to the needs of users which in turn compromises public safety. As the number of drug related harms and number of deaths continue to rise, so does the need to respond in a way consistent with harm reduction approaches that seek to ameliorate the harmful effects of drug use and stigma. Objective education about addiction and treatment through a public health paradigm could go a long way in reducing stigma and expanding treatment services (Palamar, 2013).

Given the lack of progress in reducing both the supply and demand of drugs over the course of the war on drugs, the time has come for a shift away from punitive policies to a more responsible and pragmatic approach where community-based treatment becomes standard practice whereby it is accessible to those in need. In closing, public opinion polls have consistently shown that the public supports treatment over arrest policies for drug related crimes. The current study adds to that body of literature by examining specific support for community-based treatment options. The results underscore the growing need for the expansion of community-based treatment, recovery housing, and harm reduction approaches to combat the crisis of addiction. Besides lives, what do we have to lose?

## Data Availability

The data are not publicly available.

## Footnotes

1 The 2018 Commonwealth Public Policy Poll was conducted by the L. Douglas Wilder School of Government and Public Affairs’ Office of Public Policy Outreach (OPPO) at Virginia Commonwealth University in partnership with the Virginia Secretaries of PublicSafety and Homeland Security and Health and Human Resources.
